# Patterns of Active Commuting to School in Spanish Preschool Children and Its Associations with Socio-Economic Factors: The PREFIT Project

**DOI:** 10.3390/ijerph182111180

**Published:** 2021-10-25

**Authors:** Manuel Herrador-Colmenero, Cristina Cadenas-Sanchez, Idoia Labayen, Adrià Muntaner-Mas, Diego Moliner-Urdiales, Gabriel Lozano-Berges, Pedro J. Benito, Manuel A. Rodríguez-Pérez, Álvaro Delgado-Alfonso, Joaquín Sanchís-Moysi, Vicente Martínez-Vizcaíno, Palma Chillón

**Affiliations:** 1 La Inmaculada Teacher Training Center, University of Granada, 18013 Granada, Spain; mhc@ugr.es; 2 PROFITH “Promoting FITness and Health through Physical Activity” Research Group, Department of Physical Education and Sport, Faculty of Sport Sciences, University of Granada, 18011 Granada, Spain; adria.muntaner@uib.es; 3 Institute for Innovation & Sustainable Development in Food Chain (IS-FOOD), Department of Health Sciences, Public University of Navarre, IdiSNA, Navarra Institute for Health Research, 31006 Pamplona, Spain; cadenas@ugr.es (C.C.-S.); idoia.labayen@unavarra.es (I.L.); 4 GICAFE “Physical Activity and Exercise Sciences Research Group” Research Group, University of Balearic Islands, 07122 Balearic Islands, Spain; 5 LIFE Research Group, Faculty of Humanities and Social Sciences, Universitat Jaume I, 12071 Castellon, Spain; dmoliner@uji.es; 6 GENUD “Growth, Exercise, NUtrition and Development” Research Group, Department of Physiatry and Nursing, Faculty of Health and Sport Sciences, University of Zaragoza, 50009 Zaragoza, Spain; glozano@unizar.es; 7 Laboratory of Exercise Physiology Research Group, Department of Health and Human Performance, Faculty of Physical Activity and Sport Sciences, Universidad Politécnica de Madrid, 28040 Madrid, Spain; pedroj.benito@upm.es; 8 Department of Education, Faculty of Education Sciences, University of Almería, 04120 Almería, Spain; manolo.rodriguez@ual.es; 9 SPORT Research Group (CTS-1024), CERNEP Research Center, University of Almería, 04120 Almería, Spain; 10 Department of Physical Education, Faculty of Education Sciences, University of Cádiz, 11519 Puerto Real, Spain; adelalf@gmail.com; 11 Research Institute of Biomedical and Health Sciences (IUIBS), Department of Physical Education, University of Las Palmas de Gran Canaria, 35001 Canary Islands, Spain; joaquin.sanchis.moysi@gmail.com; 12 Health and Social Research Center, Universidad de Castilla-La Mancha, 16002 Cuenca, Spain; Vicente.Martinez@uclm.es; 13 Facultad de Ciencias de la Salud, Universidad Autónoma de Chile, Talca 3460000, Chile

**Keywords:** active transportation, physical activity, determinants, family, kindergarden, motor activity

## Abstract

The aims of this study were to describe patterns of active commuting to school (ACS) of preschool children, and to analyse the relationship between ACS and family socio-economic factors. A total of 2636 families of preschoolers (3-to-5 years old) were asked to complete a questionnaire at home about the mode of commuting to school of their children and marital status, educational level, and profession of both father and mother. Chi-square analyses were applied to compare ACS between school grades and gender of the children. To analyse the association of ACS with socio-economic factors, logistic regression analyses were performed. Almost 50% of participants reported ACS of their offspring, with a higher rate in 3rd preprimary grade (5 years old) than in 1st and 2nd preprimary grades (3- and 4-years old. All, *p* < 0.05). Those preschool children who had parents with lower educational level and no managerial work had higher odds to ACS than those who had parents with higher educational level and managerial work (all, *p* ≤ 0.001). Around half of the Spanish preschool children included in this study commuted actively to school and families with lower educational levels or worse employment situation were related to active commuting to school.

## 1. Introduction

Physical activity in preschool children has shown positive health benefits such as better physical fitness, psychosocial health, cardiometabolic health, and bone and skeletal health [1,2]. Additionally, physical activity in these early ages has been associated with developmental benefits such as improved motor and cognitive development [1]. However, the prevalence of children under 6 years old who meet physical activity recommendations is low. For instance, a study in the United Kingdom reported that around 90% of the children aged 2 to 4 years old did not reach the 180 min of daily physical activity recommended by the guidelines of this country [3]. In other study developed in the Czech Republic with children aged 4 to 7 years old, around 50% of the participant did not meet the 11,500 steps count per day recommended [4]. In Spain, around a 30% of the children aged 2 to 8 years old did not meet the European physical activity recommendations (60 min of daily moderate to vigorous physical activity) [5]. However, guidelines usually did not include information about two important movement behaviours: sleep and light physical activity [6]. A novel framework to optimize health of preschool children suggests understanding the movement behaviour as a continuum in a 24 h period, in which this continuum is composed of physical activity time, sedentary behaviours, and sleep. Despite the need for targeting in all the components to obtain the greater benefit, reallocating sedentary behaviours into light physical activity might provide positive health benefits [6].

Active commuting to school is considered as a source of light and/or moderate physical activity for preschool children. This behaviour, which is defined as the use of body movement for commuting purposes (mainly walking and cycling) to cover the route between home and school, has been widely studied in children and adolescents. This daily behaviour has shown several benefits, among others it has been reported an increase in physical activity [7], better cardiorespiratory fitness when cycling [8,9], and better academic skills [10]. In Spain, around 50–60% of the children and adolescents reported active commuting to school [11,12,13]. Regarding the preschool children population, there is a low number of studies reporting the mode of commuting. Around 60% of Canadian preschool children commuted actively to school (i.e., 56% walking, 2% cycling, and 2% used a scooter), while a 1.7% used strollers or wagon and 0.4% used bike seat [14]. In some Europe countries, between 6 and 40% of preschool children (i.e., aged 2 to 6 years old) commuted actively to school, such as Italy, Estonia, Cyprus, Belgium, Sweden, Germany, and Hungary, while in others such as Spain, reported an active commuting to school in the 54% of the preschool children aged 2 to 6 years old [15]. A recent project focused on Spanish preschool children aged 4 to 6 years old showed that 46% of the children commuted actively to school, and 48% commuted actively from school [16,17]. However, these studies are not geographically distributed across Spain.

Several key factors affecting active commuting to school behaviour have been documented for children and adolescents, where distance [18,19], built environment [20,21], barriers perceived by parents [12,22], and socio-economic factors [21,23] are the most relevant. Previous studies revealed the importance of the built environment, where several factors could affect the home-to-school daily trips [24], and the walkable distance (i.e., the distance that student accept to commute on foot to school) is highlighted to ensure the effectiveness of the researches [19]. Additionally, the parental negative concerns about built environment and safety act as potential barriers for active commuting to school behaviour [25,26]. Regarding family socio-economic factors, a previous study focused in Spanish children aged 6–12 years old, concluded that families with both parents in an unemployment situation was associated with active commuting to school of their children [23]. In another study with Canadian children (aged 6–11 years old), families with parents working longer time are associated with less active commuting to school of their children [27]. Concerning parental education, a recent systematic review focused on North American children showed that a higher educational level of the parents is associated with passive commuting behaviours [28]. Previous studies have found that families with social disadvantages and low or medium-low socioeconomic levels are associated with active commuting to school of their preschool children [14,16]. However, this information is limited for preschool children, although the findings are in the same direction as findings reported in older children.

Therefore, active commuting to school behaviour might be a new venue to reallocate sedentary time (i.e., passive commuting to school) into a light and/or moderate physical activity (i.e., active commuting to school) for preschool children. However, understanding which family socio-economic factors are the most relevant to determine this behaviour will be essential to encourage active commuting to school in preschool children through appropriate promotion programs focused on families. Therefore, the aims of this study were to describe the patterns of the mode of commuting to school of preschool children, to examine the relationship between age and gender with active commuting to school among preschool children, and to analyse the relationship between this behaviour and family socio-economic factors.

## 2. Materials and Methods

### 2.1. Study Design and Participants

The current cross-sectional study is part of the multicentre project PREFIT (Assessing FITness in PREschoolers, http://profith.ugr.es/prefit, accessed on 20 October 2021), which is mainly focused on the assessment of anthropometry and physical fitness in preschool children. The PREFIT project is geographically distributed across 10 different Spain cities: Almería, Cádiz, Castellón, Cuenca, Granada, Las Palmas de Gran Canaria, Madrid, Palma, Zaragoza, and Vitoria-Gasteiz [29,30,31]. In this framework, a convenience sample of 4338 families of preschool children aged between 3 and 5 years old were invited to participate in the study. To be included in the present study, participants have to meet the following inclusion criteria: (a) to have children aged 3 to 5 years old, (b) to have complete data on family socio-economic data (i.e., marital status, educational level, and profession), (c) to have complete data on active commuting to school variables (i.e., mode of commuting and time for commuting), and (d) not to report a combined mode for commuting to school.

The data collection was conducted from January 2014 to November 2015. After received the written informed consent from the parents, they were instructed to complete an ad hoc questionnaire. Each participant (i.e., parent, mother or parental guardian) fulfilled the questionnaire at home, being returned to the research team within the next 10 days. The questionnaire consists of questions about the mode of commuting to school of the children, time for commuting to school, and marital status, educational level, and profession of both father and mother. Data about age, gender, and grade (i.e., 1st grade, 2nd grade, and 3rd grade of the second cycle in the pre-primary education, corresponding to 3, 4 and 5 years old) of the preschool children were registered in school settings.

Written informed consent was required to one parent or legal guardian to be involved in the study. The study protocol was performed meeting the ethical standards (Declaration of Helsinki revised in 2013) and the Review Committee for Research Involving Human Subjects of the University of Granada approved it (Case no. 845).

### 2.2. Measurements

#### 2.2.1. Commuting to School

Mode of commuting to school of the preschool children was assessed with the question, “How do you take your child to school?” The response options were: walking with my child, walking with my child in a baby car, by bike with a baby chair, by car, by bus/train, by motorbike, and others (where the mode was required). The mode of commuting to school of the preschool children was categorized as children and adult active (i.e., walking with my child), children and adult passive (i.e., by car, by bus/train, and by motorbike), and children passive and adult active (i.e., walking with my child in baby car and by bike with baby chair).

Additionally, for the main analyses the variable active commuting was computed, where the response option walking with my child was categorized as active commuting to school and walking with my child in baby car, by bike with baby chair, by car, by bus/train, and by motorbike response options were categorized as passive commuting to school. For both variables created (i.e., mode of commuting and active commuting), the response option others was included in the appropriate category, as long as they specified the mode used, but if not this response was excluded from the analysis. Moreover, those responses in which participants selected one active mode and one passive mode (i.e., a combined mode of commuting) were also omitted due to the impossibility to classify them as active or passive commuting to school.

Regarding the commuting time, participants self-report the time spent in commuting to school with the question “How long do you take from home to school?” The response options were less than 10 min, 10–15 min, 16–20 min, 21–30 min, and more than 30 min. The response options were categorized as ≤15 min (i.e., less than 10 min and 10–15 min) and >15 min (i.e., 16–20 min, 21–30 min, and more than 30 min).

#### 2.2.2. Family Socio-Economic Factors

Marital status was self-reported by the parents with the question “What is your current marital status?” The response options were: single, married, divorced, and widowed. The marital status was finally categorized as single (i.e., single, divorced, or widowed) or married (i.e., married).

Both parents were separately asked to self-report their highest educational level achieved, with the question “What is the father/mother’s highest educational level achieved?” The response options were: no studies, primary degree, secondary degree, bachelor, professional training, and university degree. A four-category variable was computed for each parent: no studies, primary, secondary/bachelor/professional training, and university.

The current occupation of both parents was self-reported from a list of 13 occupations, which was adapted from the Spanish National Health survey 2006 [32]. The response options were categorized in the managerial, skilled worker, and unskilled worker/unemployed.

### 2.3. Statistical Analyses

Descriptive statistics were used to analyse the characteristics of preschool children, mode and time for commuting to school, and family socio-economic factors of the preschool children. Frequencies were used for categorical variables, while the mean and standard deviation was used for continuous variables. Additionally, in order to analyse differences in age, gender, grade, and socio-economic factors between active and passive commuting to school, chi-square analyses for categorical variables and Student-T test for continuous variables were used. Finally, the chi-square test was used to assess the association between active commuting to school and age and gender of the preschool children.

In order to analyse the association of active commuting to school with personal data and socio-economic factors, logistic regression models were estimated in which active commuting to school was included as the dependent variable and age, gender, grade, commuting time, and socio-economic factors were included as independent variables in separate models. Age, gender, and commuting time were used as confounders. Interaction of age and gender with socio-economic factors were analysed. Additionally, due to the hierarchical nature of the data (children nested in schools nested in cities), a multilevel logistic regression model was estimated. However, in the school and city levels of the multilevel logistic regression model, the explanation of the unexplained variance was low and the Wald test was *p* > 0.05. Therefore, only logistic regression analyses were conducted for this study.

All analyses were conducted using IBM SPSS Statistics for Windows version 22.0 (IBM Corp, Armonk, NY, USA). The level of significance was set at *p* < 0.05.

## 3. Results

**Figure 1:**
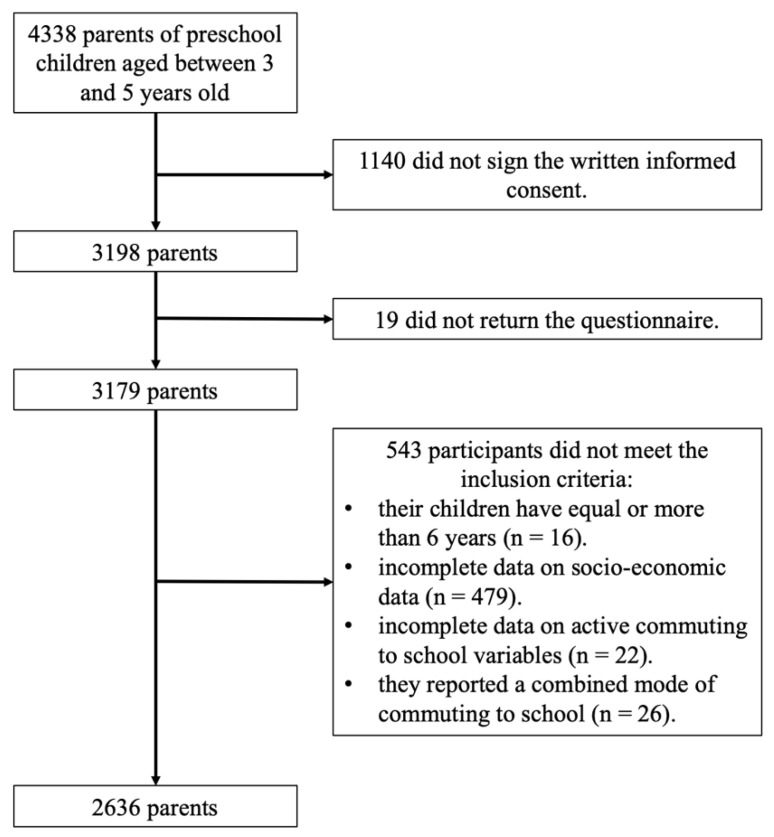
Flowchart of the study sample.

From of 4338 families of preschool children aged between 3 and 5 years old invited to participate in the study, 1140 parents declined to enroll their children in the study, with an additional 19 parents providing incomplete survey responses. A sample of 3179 participants was enrolled in the PREFIT project. From the total of 3179 participants, 16 participants were excluded because their children have equal or more than 6 years, 479 participants were excluded due to incomplete data on socio-economic data, 22 participants were excluded due to incomplete data on active commuting to school variables, and 26 participants were excluded because they reported a combined mode of commuting to school. Therefore, the final sample size was 2636 parents of preschool children aged between 3 and 5 years old (see Figure 1).

Descriptive characteristics of the preschool children and family socio-economic factors, depicted by active and passive commuting to school, are shown in Table 1. Preschool children mean age was 4.57 ± 0.87 years old, being girls almost the half of the sample (47.2%), and distributed equally along with school grades (29.7%, 34.8%, and 35.5% for 1st, 2nd, and 3rd grades respectively).

**Figure 2:**
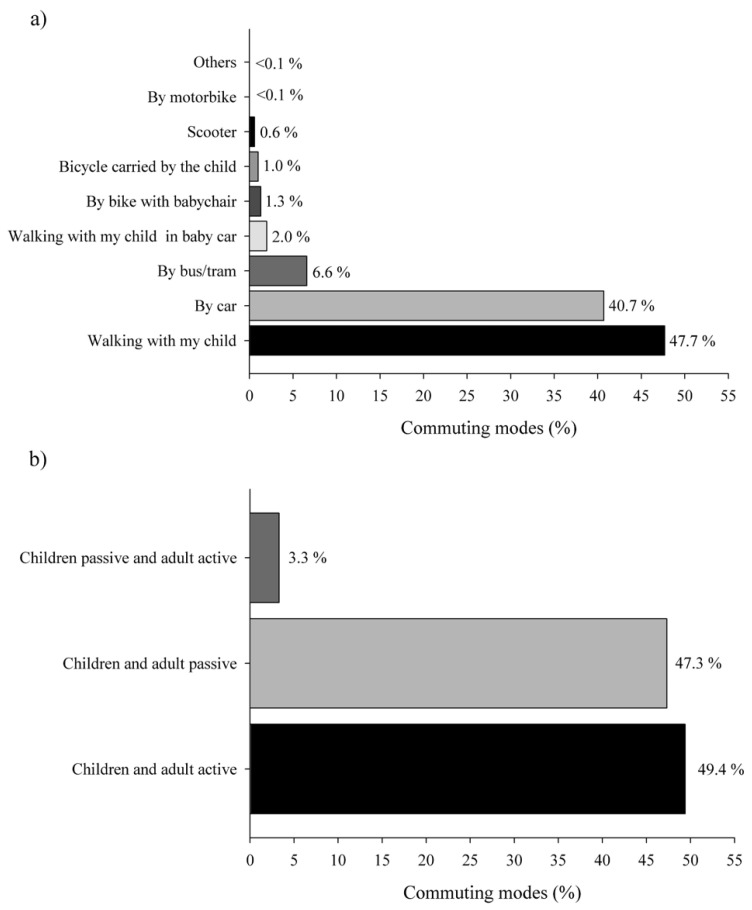
Prevalence (%) of the mode of commuting to school by response option (plot **a**) and by active and passive commuting (plot **b**). *n* = 2636 participants.

Regarding the mode of commuting to school of their preschool children (see Figure 2a), the modes more prevalent were walking to school with their child (1258 parents) and the use of a car (1072 parents), followed by the use of public transport as bus or train (175 parents). Modes of commuting to school less prevalent were walking to school with their child in a baby car, to bike with a baby chair, motorbike, and others (54, 33, 1, and 43 parents respectively). Analysing the response option others, the use of bikes carried out by children and scooter were usual (27 and 15 parents respectively). Therefore, parents and preschool children shared active or passive modes of commuting (96.7%; see Figure 2b), while responses of passive children with active parents were rare (3.3%).

Significant differences between preschool children who commute actively and passively to school were found in the commuting time (see Table 1), being more reported to use ≤15 min by those who commute actively (*p* < 0.001). Regarding family socio-economic factors, no associations were found for marital status (*p* > 0.05), while the lower educational level of the parents and unskilled work or unemployment were more reported by those parents of children who commute actively (all, *p* < 0.001).

**Figure 3:**
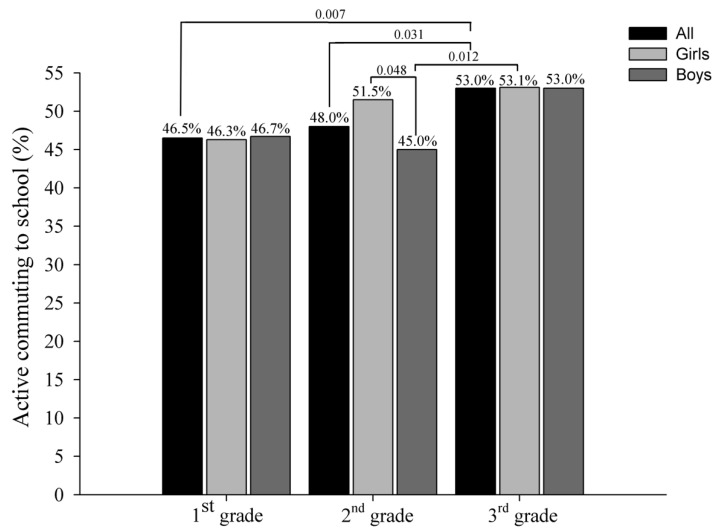
Active commuting to school by pre-primary education grade and gender.

Differences in active commuting to school between school grade and by gender are shown in Figure 3. Significant differences were found between 1st grade and 2nd grade with 3rd grade (all, *p* < 0.05), being more active the older preschool children. Analysing these results separated by gender, only a significant difference between 2nd grade with 3rd grade was found in boys (*p* < 0.05), although borderline differences were found between 1st grade with 3rd grade for both girls and boys (*p* = 0.053 and *p* = 0.058 respectively). Finally, examining the gender differences in each school grade, only a significant difference between girls and boys was found in 2nd grade (*p* < 0.05), being more active in the girls.

Associations between active commuting to school with age, gender, and grade of the preschool children and socio-economic factors of the family are shown in Table 2. Those preschool children who were older had higher odds to active commuting to school than their younger counterparts (OR = 1.213, *p* < 0.001), and those who commuted ≤15 min were three times more active commuting to school than those who commuted >15 min (OR = 3.156, *p* < 0.001). Regarding educational level, those preschool children who had parents with Primary or Secondary, Bachelor, or Professional training had higher odds of active commuting to school than those preschool children who had parents with University degree (OR between 1.345 and 2.487; all, *p* ≤ 0.001). Finally, those preschool children who had fathers who were skilled or unskilled workers/unemployed had higher odds of active commuting to school than those preschool children who had fathers with a managerial work (OR = 1.472 and 2.389 respectively, all *p* < 0.01). Those preschool children who had mothers who were unskilled workers/unemployed were around twice more active commuters to school than those preschool children who had mothers with a managerial work (OR = 2.192, *p* < 0.001). No interactions of age and gender with socio-economic variables were found (*p* > 0.05).

## 4. Discussion

The description of the mode of commuting to school of this sample of Spanish preschool children and its relationship with family socio-economic factors were analysed in this study. Overall, almost half of the included participants reported an active mode of commuting to school of their offspring, being more active the older preschool children. Moreover, active commuting to school in preschool children has been associated with less commuting time, and lower educational level and no managerial work of their parents.

In the current study, around 50% of the parents of preschool children aged between 3 and 5 years old reported an active mode of commuting to school, mainly walking. This rate of active commuting to school is lower compared with the 60% of Canadians preschool children who commuted actively [14], but higher than 20% of the preschool children (aged 3–6 years old) from China [33], the 28% from Brazilian children aged 3–5 years old [34], or the 6–40% of the preschool children (aged 2 to <6 years old) from other European countries [15]. On the other hand, the current results are similar to most of the previously reported findings in other Spanish cohorts of preschool children (aged 2–7 years old), which ranged between 46–54% [15,16,17]; although our results are lower than the 78% of active commuters reported by Terrón-Perez et al. in preschool children aged 3–5 years old [35]. The differences in the prevalence of Spanish preschool children who commute actively among studies could be affected by the differences in the sample sizes and geographical location. Moreover, these results found in Spanish preschool children, compared with the other countries, could be affected by the urban distribution of schools in each neighborhood, reducing the distances between home and schools. Findings of the current study support that living closer to the school is associated with active commuting to school. These results are in line with the previous findings in preschool [34,35] and school aged-children [28,36]. Therefore, to ensure the success of the promotion programs focused on active commuting to school among preschool children, parents should be encouraged to choose a school close to their home.

In the relationship between age and gender with active commuting to school among preschool children, our results showed that older preschool children are more active commuter to school than their younger counterparts; while, overall, no gender differences were found in this population (except in 2nd grade). Despite active commuting to school differences by age have been reported previously in school age-children [11,28], no studies were found focused on preschool children. Parents are who decide the mode of commuting in these ages, and a greater parental barrier perception has been associated with less active commuting to school [35]. Therefore, a greater perception of barriers to commute actively to school could be perceived by parents of preschool children. Additionally, the lack of gender differences found in our study is in agreement with a previous study in which they did not find a relationship between gender and walking to school [37]. A recent systematic review focused on school age-children, active commuting to school showed a weak association between active commuting to school and gender [28]. Thus, social stereotypes and constructs of the “female weakness” in children seem not to be still influencing this behavior at these early ages [38,39]. Hence, in the promotion of active commuting to school in preschool children, it seems that the age might more determinant than the gender.

In the relationship between the active commuting to school with family socio-economic factors, this study showed that active commuting to school in preschool children is higher in lower educational levels and with no managerial work of both father and mother. The current results are in line with previous studies focused on preschool children, which showed an increase of active commuting to school in lower parents’ educational level [34,37]. However, no studies have been found to compare the current results of the parental profession in preschool children. Rodriguez-López et al. [23] found that those school-age children with unemployed mothers were more likely to commute actively to school, and Brophy et al. [37] found in preschool children a relationship between active commuting to school and family income. Thus, previous findings seem to agree with the recent results. The family is a key factor in the mode of commuting of their younger members, and a less favorable family situation (i.e., lower educational level or worse employment situation) contributes to a more active commuting to school. In this sense, it could be hypothesized that the lack of economic resources in these families might avoid the use of motorized modes of transport, and more “free time” due to unemployment situations might facilitate the active commuting to school. Following the socioecological framework [40], when policy programs or educational promotion projects are developed to encourage active commuting to school behaviors, they should take into account at the interpersonal level that families with a more favorable situation (i.e., higher educational level or better employment situation) could be the population in which more effects might have the intervention.

Active commuting to school strategies for preschool children population should be encouraged from the personal level up to the policy level [41]. Taking into account the importance of the age in this behavior (located on the personal level), the educational initiatives should be focused at the interpersonal level, specifically, in the families. A single-level intervention to promote active commuting to school in preschool children might be insufficient to achieve a behavior change due to the complexity of the behavior [41]. Thus, as a first step, it might be important for parents to be aware of the benefits of the active commuting to school behavior in preschool children, such as its association with better body composition or a better physical fitness level because of the increase of a daily step count [42,43], and its influence in the health later in live [44]. After this parental sensibilization, educational strategies for preschool children and their parents throught intervention programs in the community, built environment modifications, and policy measure at school level might be introduced.

This study has several limitations and strengths. The sample recruitment could not guarantee representativeness for the country (but geographically distributed). On the other hand, the mode of commuting of preschool children was not assessed objectively, being a potential bias. Additionally, a significant number of potential participants (*n* = 1140 parents, 26% of the parent invited to participate) were missing due to refusal to enroll in this study. The lack of information about the characteristics of these parents do not allow us to explain the possible refusal reason. In other way, 479 parents (11% of the parent invited to participate) do not report socioeconomic data, being a possible reason the feeling to share personal information not necessary for the study. These unexplained drop out are a potential bias that suggest to generalize the findings with caution. However, the large sample size and its distribution around the country ensure the limitation of the effect of the local context-related characteristics. Moreover, the active commuting to school behavior has been widely studied in children and adolescents, but not in preschool children.

## 5. Conclusions

This study suggests that around half of the analyzed Spanish preschool children commuted actively to school, being the age related with this healthy behavior. Also, parental educational and employment status appears as a key factor to understand active commuting to school of their preschool children in Spain. These relationships should be tested in other context and countries to stablish a clear knowledge about active commuting to school in this age group. Moreover, researchers, school staffs, or public policies which aimed to develop multilevel strategies to promote active commuting to school behavior, should consider these results when educational programs are developed for families.

## Figures and Tables

**Table 1:** Descriptive characteristics of preschool children and socio-economic variables, split by active commuting status.

	All *n* = 2636	ACS *n* = 1301	No-ACS *n* = 1335	*p*-Value
Age, mean (SD)	4.57 (0.87)	4.64 (0.86)	4.50 (0.87)	<0.001
Gender, % (*n*)				0.274
Female	47.2 (1244)	48.3 (628)	46.1 (616)	
Male	52.8 (1392)	51.7 (673)	53.9 (719)	
Commuting time, % (*n*)				<0.001
≤15 min	82.7 (2179)	90.5 (1177)	75.1 (1002)	
>15 min	17.3 (457)	9.5 (124)	24.9 (333)	
Grade, % (*n*)				0.016
1st grade	29.7 (783)	28.0 (364)	31.4 (419)	
2nd grade	34.8 (918)	33.9 (441)	35.7 (477)	
3rd grade	35.5 (935)	38.1 (496)	32.9 (439)	
Marital Status, % (*n*)				0.737
Single	20.4 (538)	20.7 (269)	20.1 (269)	
Married	79.6 (2098)	79.3 (1032)	79.9 (1066)	
Father educational level, % (*n*)				<0.001
No studies	0.1 (3)	0.2 (3)	0.0 (0)	
Primary	9.6 (252)	11.6 (151)	7.6 (101)	
Secondary/Bachelor/Professional training	28.1 (740)	30.5 (397)	25.7 (343)	
University	62.2 (1641)	57.6 (750)	66.7 (891)	
Mother educational level, % (*n*)				<0.001
No studies	0.0 (0)	0.0 (0)	0.0 (0)	
Primary	6.0 (158)	8.2 (107)	3.8 (51)	
Secondary/Bachelor/Professional training	22.4 (591)	26.4 (343)	18.6 (248)	
University	71.6 (1887)	65.4 (851)	77.6 (1036)	
Father profession, % (*n*)				<0.001
Managerial	11.6 (307)	9.1 (118)	14.2 (189)	
Skilled worker	69.2 (1824)	67.4 (877)	70.9 (947)	
Unskilled worker/Unemployed	19.2 (505)	23.5 (306)	14.9 (199)	
Mother profession, % (*n*)				<0.001
Managerial	7.9 (209)	7.0 (91)	8.8 (118)	
Skilled worker	60.3 (1589)	53.7 (698)	66.8 (891)	
Unskilled worker/Unemployed	31.8 (838)	39.4 (512)	24.4 (326)	

SD, Standard Deviation; ACS, Active commuting to school; No-ACS, Passive commuting to school.

**Table 2:** Associations between active commuting to school and socio-economic factors.

	*n*	Active Commuting to School		
		OR	95% CI	*p*-Value
Age ^a^	2636	1.213	1.109–1.328	<0.001
Gender ^b^				
Male	1392	1	Reference	
Female	1244	1.094	0.935–1.280	0.262
Commuting time ^c^				
>15 min	457	1	Reference	
≤15 min	2179	3.156	2.524–3.947	<0.001
Grade ^a^				
1st grade	783	1	Reference	
2nd grade	918	1.070	0.880–1.300	0.498
3rd grade	935	1.313	1.081–1.595	0.006
Marital Status				
Single	538	1	Reference	
Married	2098	0.925	0.761–1.124	0.430
Father educational level				
University	1641	1	Reference	
Primary	252	1.764	1.336–2.328	<0.001
Secondary/Bachelor/Professional training	740	1.345	1.125–1.608	0.001
Mother educational level				
University	1887	1	Reference	
Primary	158	2.487	1.744–3.547	<0.001
Secondary/Bachelor/Professional training	591	1.628	1.345–1.972	<0.001
Father profession				
Managerial	307	1	Reference	
Skilled worker	1824	1.472	1.143–1.896	0.003
Unskilled worker/Unemployed	505	2.389	1.773–3.220	<0.001
Mother profession				
Managerial	209	1	Reference	
Skilled worker	1589	1.077	0.801–1.449	0.624
Unskilled worker/Unemployed	838	2.192	1.601–3.000	<0.001

^a^ Adjusted only by gender and commuting time. ^b^ Adjusted only by age and commuting time. ^c^ Adjusted only by age and gender. All analyses were adjusted by age, gender, and commuting time. OR, Odds Ratio; CI, Confidence Interval.
